# Winter Climate of the Nile

**Published:** 1890-03-01

**Authors:** 


### CLIMATOLOGY.

### WINTER CLIMATE OF THE NILE.

Dr. Savill sums up an article in the *Lancet* on "The Winter Climate of the Nile," as follows: "It may be summarily described as exceedingly dry, sunny, and mild, the air having a far more bracing property than is to be found in most warm atmospheric conditions." There are, however, disadvantages. There is a wide, diurnal range, and the nights are often very cold. Then there is the *khamseen*, or desert wind, which blows from the south or south-west, and scorches like a furnace-blast. It also brings up clouds of fine dust. It generally blows for three days at a time, after an interval of three to five days, and it commences first in February. The cases most benefited are incipient phthisis and rheumatism, the dryness being especially suitable to the latter. "The martyr to chronic rheumatism finds a veritable elysium in Upper Egypt." The clothing best suited is that worn during an English spring, but no one should be without a warm overcoat for chilly evenings. Nile water should not be drunk unless boiled and filtered, otherwise it may cause diarrhœa.

